# Cross-Cultural Adaptation and Validation of Social–Emotional Questionnaires in Danish

**DOI:** 10.3390/audiolres15050133

**Published:** 2025-10-09

**Authors:** Abigail Anne Kressner, David Harbo Jordell, Filip Rønne

**Affiliations:** 1 Copenhagen Hearing & Balance Centre, Department of Otorhinolaryngology, Head and Neck Surgery & Audiology, Rigshospitalet, Technical University Hospital of Greater Copenhagen, 2100 Copenhagen, Denmark; 2 Clinical and Technical Audiology, Hearing Systems Section, Department of Health Technology, Technical University of Denmark, 2800 Kgs. Lyngby, Denmark; 3 ORCA Labs, WS Audiology, 3540 Lynge, Denmark

**Keywords:** patient-reported outcome measures, questionnaires, hearing loss, social participation, social isolation

## Abstract

**Background/Objectives**: This study aimed to linguistically and culturally adapt the Social Participation Restrictions Questionnaire (SPaRQ) and the Hearing Handicap Inventory (HHI) for the Elderly/Adults to Danish and to investigate the reliability and validity of the questionnaires and their subscales in a clinical population. These questionnaires are quantifiable self-assessment tools that are used internationally to evaluate the social–emotional impacts of hearing impairment. **Methods**: The translation and cross-cultural adaptation procedures followed recommendations to adapt hearing-related questionnaires for different languages and cultures. In total, 64 participants (43 hearing aid users and 21 hearing aid candidates) completed both questionnaires using a test–retest paradigm. **Results**: Reliability analysis showed good internal consistency (Cronbach’s alpha between 0.82 and 0.94) and good agreement between the test and retest rounds (intraclass correlation values between 0.79 and 0.88) with both questionnaires. Neither SPaRQ nor HHI were correlated with better-ear PTA. However, SPaRQ and HHI, as well as their subscales, were significantly correlated with each other. Significant differences were observed at baseline between the HA users and candidates in terms of the better-ear PTA, but the distributions of subscale scores were broad and overlapping. **Conclusions**: The Danish version of SPaRQ is a reliable instrument for measuring the subjective impacts of hearing impairment. It can be used to capture the experiential aspects of hearing impairment that are not necessarily captured with objective measures of hearing.

## 1. Introduction

Patient-reported outcome measures (PROMs) are increasingly recognized as essential tools in both clinical and research settings for capturing the subjective experiences of individuals with health conditions. In audiology, PROMs provide valuable insights into how hearing loss affects daily life, emotional well-being, and social functioning—factors that are not always reflected in objective audiometric assessments [1]. Standardized questionnaires offer a structured and quantifiable means of assessing these impacts, supporting more comprehensive diagnosis, treatment planning, and evaluation of intervention outcomes. Many such instruments have been employed to assess the perceived emotional, social, and behavioral consequences of hearing impairment (e.g., [2,3,4,5]). However, to be useful across diverse populations, these tools must be linguistically and culturally adapted and validated for the target context [6].

The Hearing Handicap Inventory for the Elderly (HHIE) and its adult counterpart (HHIA) are among the most widely used patient-reported outcome measures in audiology. These questionnaires assess the perceived emotional and social consequences of hearing loss and have been translated and validated in numerous languages, making them a cornerstone in both clinical and research contexts [7,8]. A recent psychometric reevaluation led to the development of a revised version of the HHI (RHHI), aimed at improving its measurement properties and clinical utility [9]. Despite the international relevance of these tools, a validated Danish version had not yet been established at the outset of this work. This represents a significant gap, as the availability of culturally and linguistically appropriate tools is essential for accurately capturing patient experiences.

While the HHI provides valuable insights into the perceived handicap associated with hearing loss, it does not fully capture the broader psychosocial dimensions that may influence or result from hearing difficulties. Psychosocial functioning—including social and emotional well-being, social participation, and self-perception—plays a critical role in how individuals experience and adapt to hearing impairment [10]. These aspects are particularly relevant given the growing recognition of the links between hearing loss and mental health outcomes, including but not limited to social isolation, depression, and reduced quality of life [2,3,11,12]. The Social Participation Restrictions Questionnaire (SPaRQ) was developed to address some of this gap by measuring the negative thoughts, feelings, and behaviors that individuals may experience in social contexts specifically due to hearing loss [13]. By focusing on psychosocial functioning, SPaRQ complements traditional measures like the HHI and offers the possibility to assess from a complementary perspective the lived experience of hearing impairment.

In light of the need for validated, culturally appropriate tools to assess the multifaceted impact of hearing loss, the current study aims to contribute to this effort by focusing on three key objectives. The first objective was to culturally adapt and translate SPaRQ and HHI for adults with hearing loss in the Danish healthcare system. In a separate parallel investigation, the psychometric properties of the HHI translation were assessed, and the questionnaire was validated [14]. Thus, the second objective of this investigation was to assess the psychometric properties of the Danish translations of SPaRQ, ensuring its reliability and validity within a Danish-speaking population. To achieve this, we employ HHI as a reference. Finally, the third objective was to assess the validity of, and the association between, the subscales of SPaRQ and HHI. Together, these efforts aim to support the broader implementation of PROMs in Danish audiological practice and research.

## 2. Materials and Methods

This study was part of a larger project focused on a longitudinal investigation of self-rated social participation and hearing handicap in HA users. Participants were asked to complete questionnaires a total of five times in the course of the study. To psychometrically evaluate the properties of the questionnaires, only the first two measurement points are assessed.

### 2.1. Questionnaires

The process suggested by Hall et al. [6] to adapt hearing-related questionnaires to different languages and cultures was employed. In the first stage of translation, three independent forward translations were produced by members of the translation committee, who each have experience in the field of audiology and in questionnaire research. They all met to consolidate and align on the translation. In the next step, two back-translators completed independent back translation to English and then met to consolidate their versions. Finally, all five committee members met to compare the translations to the original questionnaires and discuss and revise any inconsistencies. None of the authors were part of the committee. A pilot test was conducted thereafter with the questionnaire translations by asking three hearing-impaired, Danish native speakers to complete the questionnaires and report any difficulties in understanding the task at hand or the questions posed. Two grammatical word choice revisions were suggested and then implemented in the final version of the questionnaires (see Appendix A and Appendix B).

The SPaRQ is a 19-item questionnaire comprising two subscales that measure social behaviors (9 items) and social perceptions (10 items) in adults with hearing loss (see Table A1 and Table A2, respectively). Every item is measured on an 11-point Likert scale (0 = completely disagree, 10 = completely agree), where higher scores suggest greater restrictions in social participation. The first question of the SPaRQ Behaviors subscale is, “Because of my hearing loss, I find it difficult to talk with staff in places such as shops, cafes, or banks.” The first question of the SPaRQ Perceptions subscale is, “Because of my hearing loss, I find social gatherings stressful.” The respondents’ raw scores are summed to produce total scores for each subscale, which follows the recommended procedure for “low stakes” studies, as detailed in the supplementary material provided when obtaining a copy of the original English questionnaire [13]. A third section of the instrument asks about HA use (Table A3). Responses to this question have not been included in the analysis for the current study.

The 25-item HHIE measures self-rated hearing handicap on two subscales: the social/situational and emotional consequences of hearing loss. The HHIA [7] includes three modified items from the HHIE [8] that were adapted to a younger (i.e., 64 years of age and less) target population to include items, for example, that refer to the workplace. The more recently developed 18-item RHHI is a subset of the HHIE and HHIA [9]. An example question from the questionnaires is, “Does a hearing problem cause you to use the phone less often than you would like?” For completeness, all 28 questions (i.e., the 22 items in common between the two plus the 6 questions not common between the two) were translated in the Danish version of the HHI (Table A4). Each item is answered on a 3-point scale (‘yes’, ‘sometimes’, and ‘no’) with a maximum possible total score of 100, where a ‘yes’ answer is given 4 points, a ‘sometimes’ answer is given 2 points, and a ‘no’ answer is given 0 points. During the translation process, the translation committee agreed that the response options for four of the items needed to be revised by adding an option to respond, “not relevant,” as these items address situations that may not be relevant for every respondent: religious ceremonies, conversations with clients, colleagues or customers, cinema, and theater. Such responses were scored in the same way as a ‘no’ answer, meaning they were given 0 points. For this study, responses were analyzed using only the RHHI set of questions [9], meaning everyone was scored with the same 18 items, none of which included a ‘not relevant’ category. The 18 questions that constitute the RHHI, as well as which questions make up each of the subscales, are indicated in Table A4. For ease of reading, we refer to the instrument going forward as HHI.

### 2.2. Participants

Participants were recruited from the Bispebjerg Audiological Clinic, a satellite clinic of the Copenhagen Hearing and Balance Center at Rigshospitalet, Copenhagen University Hospital. However, participants had different recruitment pathways and inclusion criteria depending on whether they were existing HA users or HA candidates. Participants were blinded to the fact that there were two groups, and the project was described in the same way to both groups—namely that the project’s purpose was to gather knowledge about the effects of HA intervention.

For the HA candidate group, we invited all new patients seen at the Bispebjerg Audiological Clinic, a satellite clinic of the Copenhagen Hearing and Balance Center at Rigshospitalet, Copenhagen University Hospital, between 21 February 2022 and 20 April 2022 to participate in the study if they (1) were 18 years or older, (2) were assessed as needing a HA, (3) had not used a HA before, (4) had decided to be fitted for a HA, (5), had access to Denmark’s platform for secure, digital post (e-Boks) and (6) expressed that filling in an online questionnaire outside of the clinic via an invitation sent through e-Boks would be possible. HA eligibility criteria vary depending on the specific etiology of the hearing loss, and eligibility in general is determined on an individual basis, but as a guideline in the case of presbycusis, HAs are offered if thresholds are worse than 25 dB at 2 kHz and above. Moreover, the HA clinic is part of the social healthcare system, so HA treatment is given at no extra cost to the individual. Interested participants were given a letter describing the study together with a form to collect their contact information and consent to be contacted at a later point in time. Thereafter, patients were contacted in person (e.g., directly after their appointment) or via phone, where they were offered a more in-depth information meeting and where details of the study could be discussed. Recruitment for the HA candidate group ceased when 50 participants enrolled or otherwise ended when the recruitment period ended.

For the HA user group, patients were invited to participate in the study if they (1) had previously been to the Bispebjerg Audiological Clinic, (2) were 18 years or older, (3) had given consent within the last 12 months to being contacted to participate in research studies, (4) had been a HA user for at least six months, (5) had no plans to obtain a new HA in the next three months, and (6) expressed that filling in an online questionnaire outside of the clinic would be possible. Specifically, a letter describing the study was sent via Denmark’s platform for secure, digital post (e-Boks), and individuals interested in participating were asked to contact us via email or phone for a more in-depth information meeting where details of the study could be discussed. Recruitment for the HA user group ceased when 50 participants enrolled or otherwise ended when the recruitment period ended.

### 2.3. Procedure

**Figure 1:**
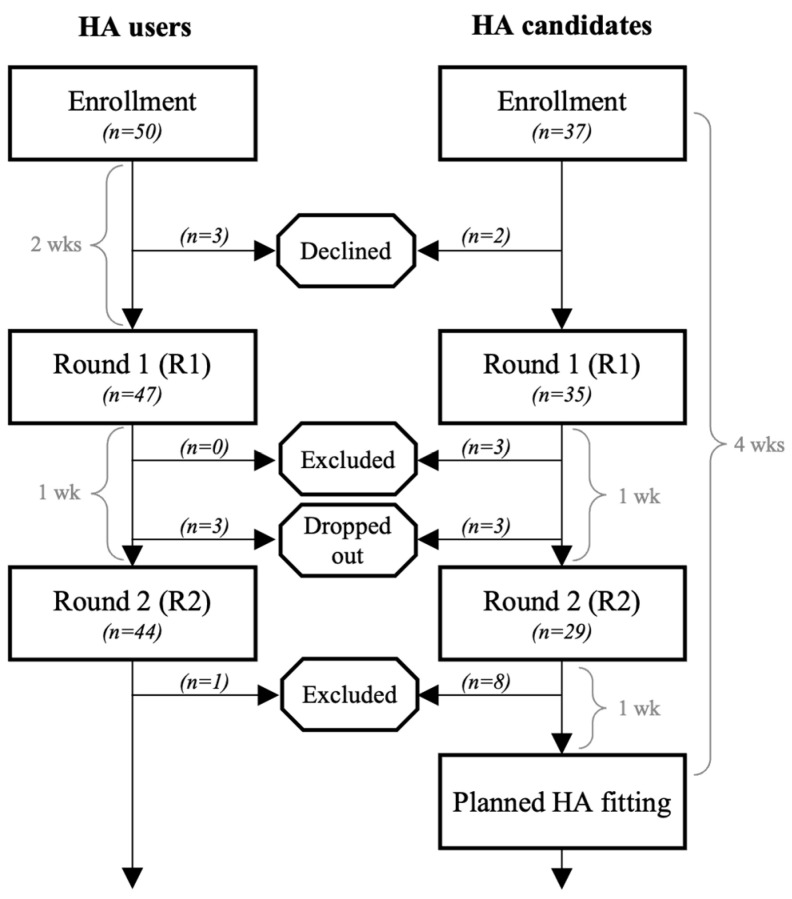
Participant flowchart visually showing enrollment, exclusion, and participation numbers in each stage. The time durations indicate how nominal data collection points were determined.

Study data were collected and managed using REDCap electronic data tools hosted at Region Hovedstaden [15]. Participants who decided to participate in the study were, as a starting point, registered in REDCap, and thereafter, a secure electronic mail was sent via e-Boks to participants to obtain informed consent digitally. Participants were then asked to fill out background information and then to fill out the questionnaires two times such that there were two data collection points timed approximately one week apart. For the HA candidate group, these two data collection points were timed individually so that they would occur just before it was planned that the participant would receive their HA. At the time of the planning of this study, participants were typically scheduled to return to the clinic to receive their HA 4 weeks after their initial consultation (Figure 1). In contrast, for the HA user group, these two data collection points were timed relative to the date of consent. The difference in the timing of these data collection points was important for the larger study to align the course of all assessments between the two groups, but the timing difference is less important in the current investigation. We will refer to these rounds of questionnaires going forward as Round 1 (R1) and Round 2 (R2).

A secure mail to collect the first round of questionnaires was sent to the participants after they gave informed consent to participate in the project. In the secure mail, participants were given brief instructions and a direct link to REDCap, and from within REDCap, participants were asked to complete the first round (R1) of SPaRQ and HHI questionnaires. All participants were sent a reminder approximately three days after the first invitation to complete the questionnaires if they had not yet completed them. This reminder came in several forms, either via e-Boks or through a text message, and in some cases, by ringing via phone. A second reminder was sent after approximately another three days, if necessary.

The timing of the second round (R2) of questionnaires was one week after the first, so for the HA candidate group, this was one week before the scheduled HA fitting, and for the HA user group, this was three weeks after they gave informed consent to participate. In the same way as the first round, participants were sent a secure mail with brief instructions and a link to REDCap. At the start of this round though (and all rounds thereafter), participants were first asked whether (1) they had any appointments at the Bispebjerg Audiological Clinic since the last time they filled out the questionnaires (yes or no), and if they answered yes, (2) they were asked what the purpose of the appointment was (delivery of a new HA; adjustment of the sound; modification, replacement, or grinding of the earmold; repair or service; instruction in use or maintenance; or other). These questions were included to ensure the participants did not actually receive an intervention between the two measurement points. They were then asked to complete the SPaRQ and HHI questionnaires. Participants were reminded to fill out the questionnaires if they had not done so following the same protocol for reminders in the first round.

At any point in time, participants could remove themselves from the study by passively stopping replies or by contacting us actively. In the latter case, the mails with links to the questionnaires, as well as the follow-up reminders, were no longer sent. Participants were always given the opportunity to retract their consent, as well as their information.

As part of the standard clinical care at the Bispebjerg Audiological Clinic, masked, audiometric thresholds were measured for the left and right ear of each participant using pure-tone threshold audiometry. Sound was presented in an isolated sound booth via either TDH39 supra-aural headphones or E-A-RTONE 3A insert earphones and a B71 bone transducer headset. Audiogram data were collected with the Interacoustics AC40 clinical audiometer, stored in a clinical database, and then transferred manually to REDCap for each individual after completion of the questionnaires.

### 2.4. Statistical Analysis

Statistical analyses were carried out using Matlab Version 24.2.0.2740171 (R2024b) Update 1 (Mathworks, Natick, MA, USA). The demographics of the participants in the study are reported based on group distributions in terms of age, sex, and better-ear PTA. Significant differences between the groups were assessed with a Wilcoxon rank-sum test (equivalent to a Mann–Whitney U test) for age and better-ear PTA and a chi-square test for age, always with a significance level of α=0.05. The psychometric properties of the questionnaires were assessed in terms of construct validity (including both convergent validity and discriminative validity), as well as internal consistency and test–retest reliability, as detailed below.

Convergent validity refers to the extent to which a questionnaire is correlated with another questionnaire. Similarly to the hypothesis that was set forth in the validation of the English version of SPaRQ [13], we hypothesized that the Danish version of SPaRQ would have a strong, positive correlation (±0.60 or above) with the Danish version of HHI. To investigate this, we assessed Spearman’s rank order correlation coefficient between the two scales and their subscales in R1.

Discriminative validity refers to the ability of a questionnaire to distinguish between patient groups [16]. We hypothesized that the HA candidate group would report more social restrictions and higher hearing handicap at baseline than the HA user group. To test this, the effect of the group (i.e., users versus candidate) on each questionnaire subscale in R1 was assessed with a Wilcoxon rank-sum test with a significance level of α=0.05. Effect sizes were estimated with Cliff’s delta, which is a non-parametric effect size measure suitable for ordinal or non-normally distributed data. Another hypothesis is that patients with a higher better-ear PTA would report more social restrictions and higher hearing handicap. To investigate this, Spearman’s rank order correlation coefficients are reported to quantify the association between better-ear PTA and the questionnaire responses in R1.

Internal consistency refers to the level of inter-relatedness among the items [17]. Cronbach’s alpha was used to assess this property based on the responses to the questionnaires in R1. Intraclass correlation (ICC) was computed for each subscale to assess the degree of absolute agreement among the measurements [18]. In addition to the estimation of ICC, a hypothesis test with a significance level of α=0.05 was performed with the null hypothesis that ICC is equal to zero. Lastly, a Wilcoxon signed rank test (α=0.05) was performed to assess whether the individual change from R1 to R2 for each participant followed a normal distribution with a mean equal to zero.

## 3. Results

In total, 87 participants were enrolled, with 50 in the HA user group and 37 in the HA candidate group. The demographics of the two groups are shown in Table 1. Eighty-two participants completed R1 (i.e., 94% of the participants enrolled in the HA user group and 95% of the participants enrolled in the HA candidate group). However, three of the participants in the HA candidate group were excluded, as they answered the R1 questionnaires after receiving their HA(s). Seventy-three participants completed R2 (i.e., 88% of the participants enrolled in the HA user group and 78% of the participants enrolled in the HA candidate group). Participants in the HA user group were excluded in R2 if they answered ‘yes’ to having had an intervention between R1 and R2, which was the case for one participant. Eight of the participants in the HA candidate group were excluded from R2, as they answered the R2 questionnaires after receiving their HA(s). Thus, a total of 64 participants were included for the remaining analysis. The participant flow is depicted visually in Figure 1.

**Figure 2:**
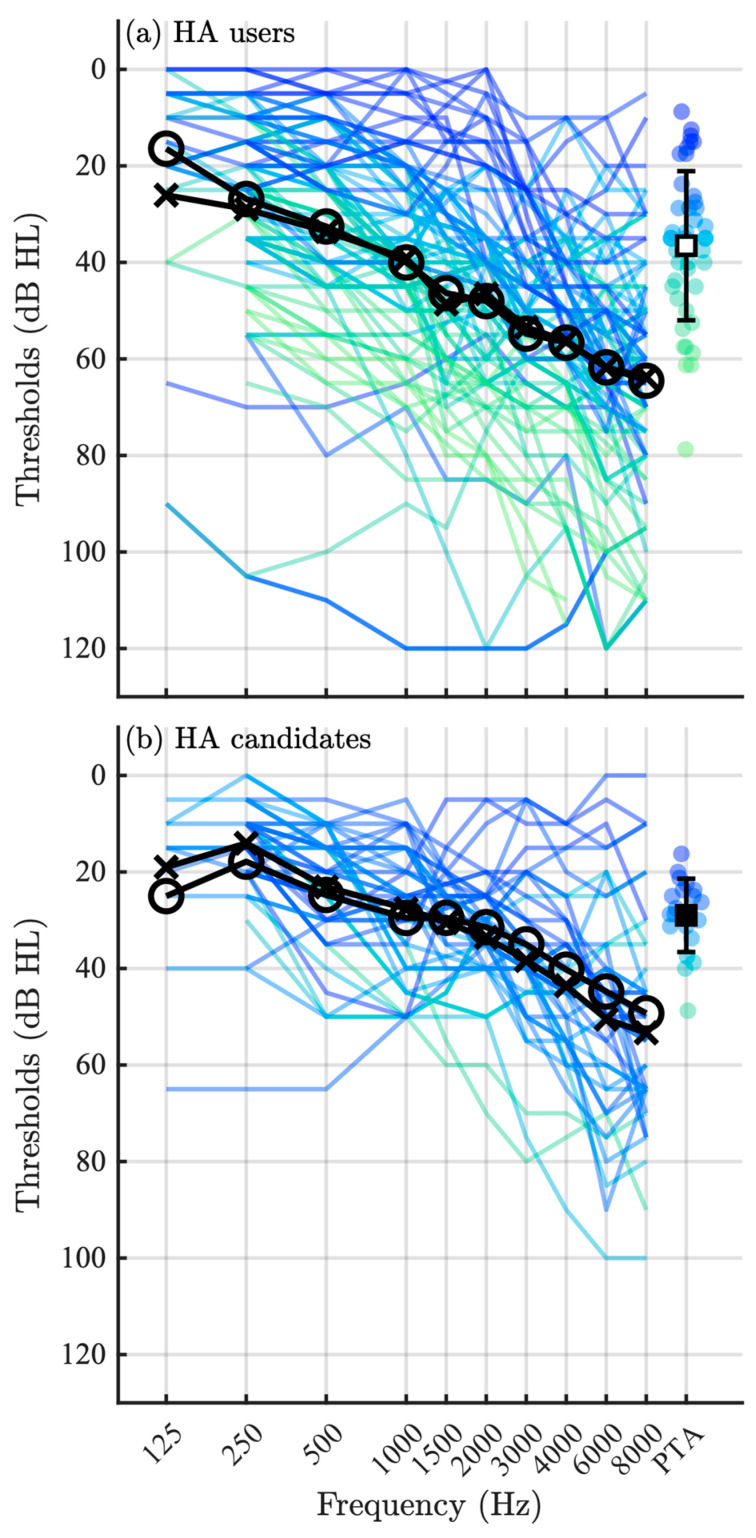
Audiograms for the (**a**) HA users and (**b**) HA candidate groups, where audiograms for both ears are shown in a single unique color (or gray tone) for each individual and the group means are shown in black. The black crosses indicate the group mean for the left ear, and the black circles indicate the group mean for the right ear. The better-ear pure-tone average (PTA) is shown for each individual on the right side of the figures, along with the mean and (SD) for the group.

Participants were, on average, 65.7 yr (SD 11.9) in the HA user group and 63.7 yr (SD 14.7) in the HA candidate group, with an approximately equal distribution of sex in each group. The age of the participants ranged from 24 to 79 yrs. Age was not significantly different between the two groups (z=0.35, p=0.73), nor was sex ($\Psi^{2}=1.79$, p=0.41). Figure 2a shows the audiograms for the HA user group, and Figure 2b those for the HA candidate group. The mean better-ear pure-tone average (PTA) (i.e., the average of thresholds at 0.5, 1, 2, and 4 kHz) for the HA user group was 36.5 dB HL (SD 15.4), whereas the mean better-ear PTA for the HA candidate group was 29.0 dB HL (SD 7.6). The distributions of better-ear PTA were significantly different between the two groups (z=2.24, p=0.03).

### 3.1. Construct Validity

SPaRQ and HHI were strongly correlated with one another ($r_{s}=0.63$, p<0.001), as were their subscales (Table 2). The SPaRQ Perceptions subscale was strongly correlated with the HHI Emotional subscale, and only moderately correlated with the HHI Social subscale. On the other hand, the SPaRQ Behaviors subscale was only moderately correlated with both the HHI Emotional subscale and the HHI Social subscale.

**Figure 3:**
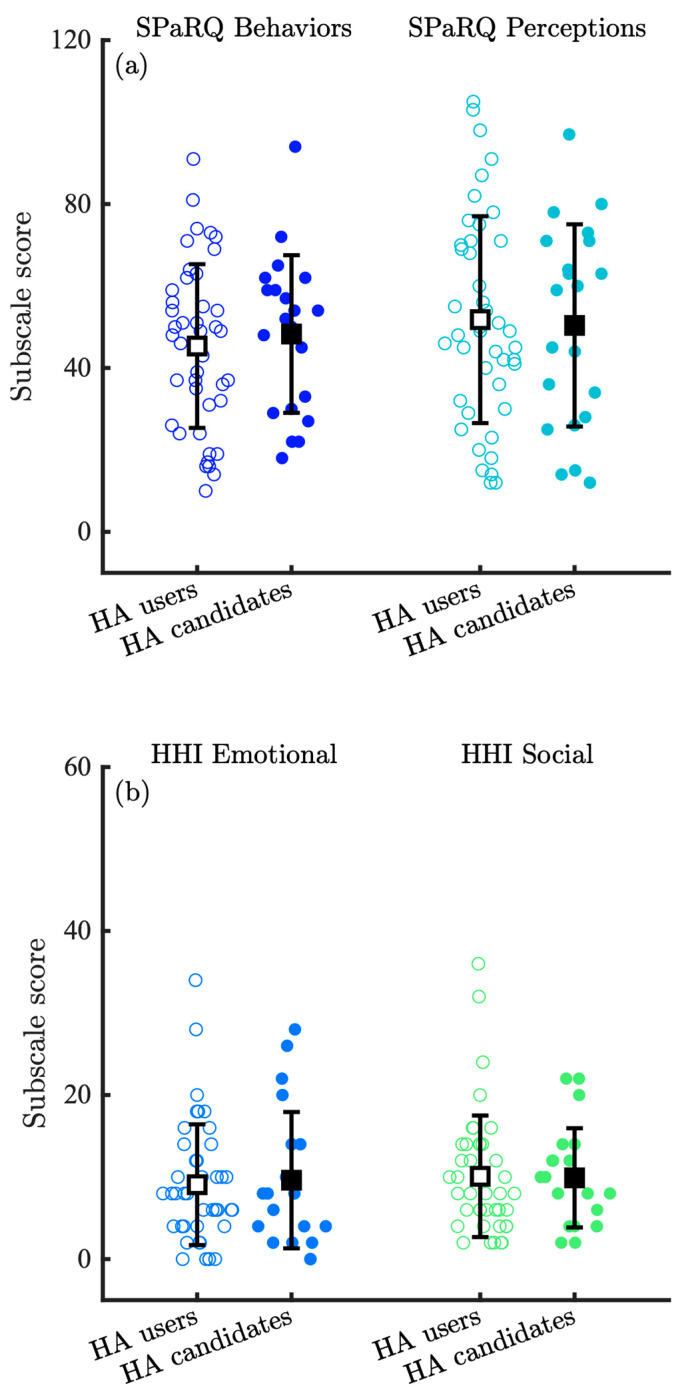
Distribution of (**a**) SPaRQ and (**b**) HHI responses at baseline (Round 1; R1) for the HA users and HA candidate groups, where individual data points are shown with colored circles, the means with black squares, and the standard deviation with black lines. Open circles and squares correspond to the HA user group, whereas filled circles and squares correspond to the HA candidate group.

Figure 3 compares the distribution of SPaRQ and HHI scores for the HA users and HA candidate groups at baseline in R1. The HA user group reported a mean score of 45.3 (SD 20.0) on the SPaRQ Behaviors subscale, and the HA candidate group reported a mean score of 48.3 (SD 19.2), revealing a significant effect of the group (z=9.97, p<0.001). The effect size was small, however, (δ=−0.09), suggesting that while there may be a statistically significant difference between the distributions, the practical difference between the groups is small. The means for the SPaRQ Perceptions subscale were also similar, with a mean score of 51.8 (SD 25.2) for the HA user group and 50.4 (SD 24.7) for the HA candidate group; the distributions were statistically different (z=9.97, p<0.001), but the effect size was small (δ=0.02). In the HHI Emotional subscale, the HA user group reported a mean score of 9.1 (SD 7.4) whereas the HA candidate group a mean score of 9.6 (SD 8.3), and again, the distributions were statistically different (z=7.23, p<0.001), but the effect size was small (δ=−0.01). In the HHI Social subscale, the HA user group reported a mean score of 10.1 (SD 7.4) whereas the HA candidate group a mean score of 9.9 (SD 6.0); furthermore, the distributions were statistically different (z=9.35, p<0.001), and the effect size was small (δ=−0.02). The overlap of these two groups at baseline was not expected, as it was hypothesized that the HA candidate group would report more social restrictions and higher hearing handicap at baseline than the HA user group.

**Figure 4:**
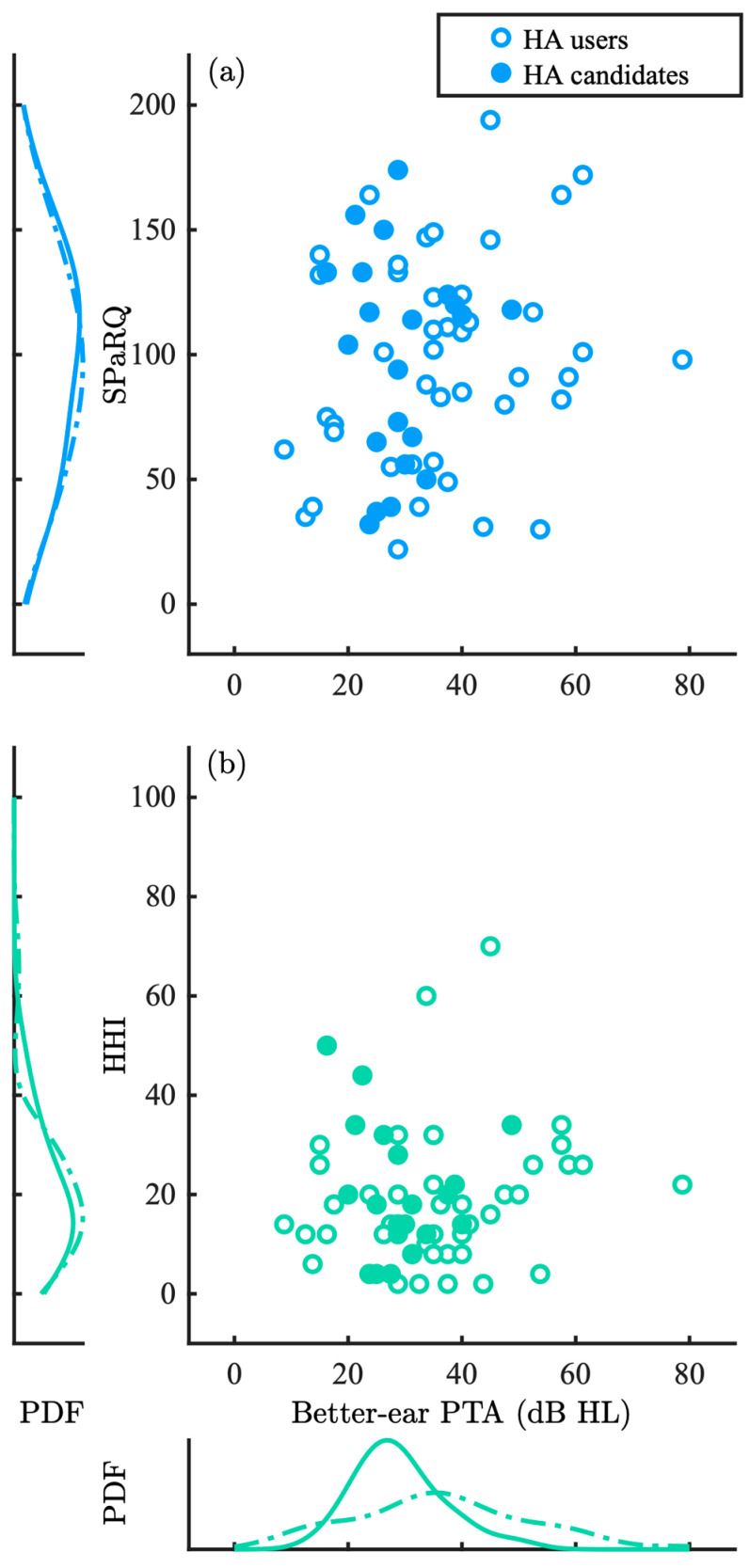
Combined (**a**) SPaRQ and (**b**) HHI scores in Round 1 (R1) as a function of better-ear PTA, where probability density functions (PDFs) for each group are estimated and shown along each axis. Individual data points are shown with open, blue circles for the HA users and filled, turquoise circles for the HA candidate groups, corresponding to dotted lines for the HA users and solid lines for the HA candidates.

Figure 4 shows total (a) SPaRQ and (b) HHI scores in R1 as a function of better-ear PTA, where probability density functions for each group are estimated and shown along each axis. SPaRQ at baseline was not correlated with better-ear PTA ($r_{s}=0.13$, p=0.29). Similarly, HHI at baseline was not correlated with better-ear PTA ($r_{s}=0.13$, p=0.30).

### 3.2. Internal Consistency and Test–Retest

**Figure 5:**
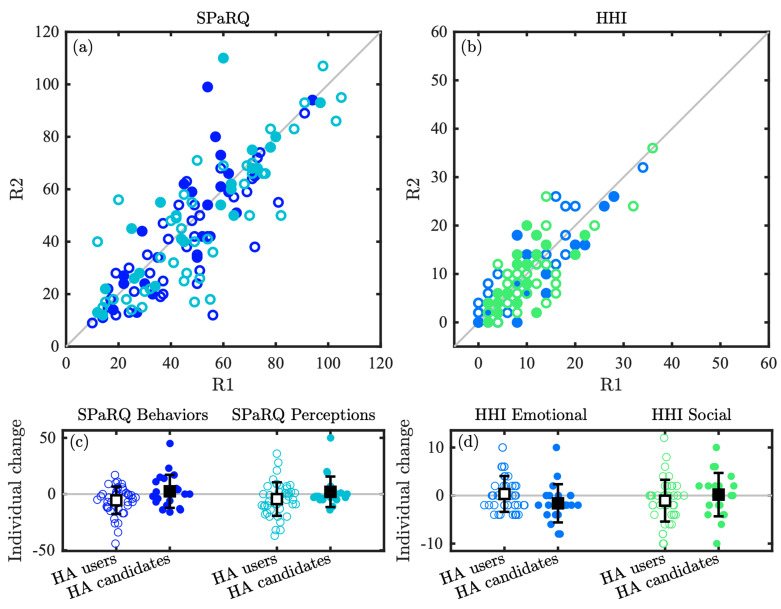
Test–retest analysis between Rounds 1 and 2 (R1 and R2), including scatter plots showing the raw scores of each subscale of the Danish (**a**) SPaRQ and (**b**) HHI questionnaires, as well as swarm charts depicting the distribution of the individual changes from R1 to R2 for each (**c**) SPaRQ and (**d**) HHI subscale. Individual data points are shown with open circles for the HA user group and filled circles for the HA candidate group, and the colors of the circles in (**a**,**b**) correspond to the respective colors used in (**c**,**d**) for each subscale. The group means are superimposed on top of the swarm charts in the black squares, and the standard deviations are shown with black lines.

Assessment of the internal consistency of the subscales in R1 is shown in Table 3, where all subscales had Cronbach’s alpha values in the target range of 0.7 to 0.95 [13]. Table 3 also shows the ICC agreement analysis, as well as the average within-subject changes from R1 to R2. All four subscales obtained good agreement (i.e., defined as ICC values between 0.75 and 0.90 [19]) and were highly significant (p<0.001). Figure 5a,b depict the variation in the individual scores on each of the SPaRQ and HHI subscales between R1 and R2, revealing good agreement between the test and retest measurements, aligning well with the ICC agreement analysis (Table 3).

Figure 5c,d show swarm charts of the amount of individual change from R1 to R2 for each participant, whereas Table 3 reports the mean and single-run standard deviation (SD) of these changes for each subscale. The observed changes were not significantly different between the rounds for the SPaRQ Behaviors subscale (z=−1.94, p=0.05), the SPaRQ Perception subscale (z=−1.94, p=0.05), the HHI Emotional subscale (z=−0.99, p=0.32), nor the HHI Social subscale (z=−1.36, p=0.17).

## 4. Discussion

This study aimed to translate and culturally adapt two widely used hearing-specific questionnaires—SPaRQ and HHI—into Danish and to evaluate their psychometric properties in a clinical population. The results suggest strong internal consistency and good test–retest reliability for both instruments. Construct validity was supported by significant correlations between the SPaRQ and HHI, as well as between their subscales, while discriminative validity was less clear, as no substantial differences in baseline scores were observed between HA users and HA candidates. However, the lack of group differences may also be interpreted not as a validity failure, but as evidence that psychosocial impact is influenced by factors beyond audiometric thresholds and HA use. Importantly, neither questionnaire showed a meaningful correlation with better-ear PTA, reinforcing the value of patient-reported outcome measures in capturing subjective experiences of hearing impairment that are not reflected in audiometric thresholds.

The validation of the Danish SPaRQ in this study aligns closely with the psychometric properties reported by Heffernan et al. [13], who originally developed and refined the questionnaire using both Rasch analysis and traditional psychometric techniques. Similarly to their findings, our results demonstrated strong internal consistency (Cronbach’s α = 0.91–0.94) and good test–retest reliability (ICC = 0.79–0.83) for both the Social Behaviors and Social Perceptions subscales. While Heffernan et al. focused on construct validity through correlations with other self-report measures, our study extended this by examining associations with audiometric thresholds. Notably, we found that SPaRQ subscales were not meaningfully correlated with better-ear PTA, reinforcing the questionnaire’s focus on subjective psychosocial experiences rather than objective hearing loss severity.

In addition to our SPaRQ results aligning with earlier work, our findings on the HHI are also consistent with earlier studies, both with regard to the original English-language version of HHI [7,8,9,20] and with the Danish-language version [14]. HHI and its related HHIE, HHIA, and screening versions have been translated and validated in many other languages as well, including Spanish [21], French [22], Italian [23], German [24], Swedish [25], Hindi [26], and Arabic [27], among others, allowing it to be used for assessing hearing-related self-perceived difficulties in different populations worldwide. The specific list of languages includes some from Europe, Asia, and South America, demonstrating its broad international application. Together, these studies confirm the robustness of the HHI across different languages, formats, and populations, and support its continued use as a benchmark for assessing hearing handicap.

The weak correlation observed between SPaRQ or HHI scores and better-ear PTA in our study reinforces the growing consensus that objective audiometric thresholds alone are insufficient to capture the lived experience of hearing loss. This finding is consistent with prior research emphasizing that hearing loss impacts not only auditory function but also suprathreshold hearing deficits and social and emotional well-being—domains that are often invisible in audiograms [1,10,21]. These results support the use of social–emotional questionnaires as complementary tools in clinical practice, particularly for capturing aspects of hearing-related quality of life that are not necessarily reflected in audiometric data. Notably, Timmer et al. [10] outline a five-step framework for integrating social–emotional well-being into audiological rehabilitation, highlighting the importance of using validated self-report tools, such as SPaRQ and HHI, to identify and monitor psychosocial impacts. Despite measurable differences in hearing thresholds, patients—irrespective of whether they were a HA candidate or user—reported a wide range of perceived social restriction and hearing handicap. This overlap suggests that psychosocial consequences of hearing loss may be influenced by factors beyond audiometric severity and HA experience, including, for example, coping strategies, social support, or psychological flexibility. Incorporating tools like SPaRQ or HHI into routine clinical practice may therefore help clinicians better tailor interventions to individual needs and promote holistic, person-centered care [2,10,11].

Several limitations of the present study should be acknowledged. First, although the final analysis included 64 participants, this falls short of the recommended minimum sample size of 100 participants for robust estimates of construct validity and internal consistency [13,28]. Moreover, the small sample size limited power to detect small effect sizes, especially for discriminative validity. While our findings are consistent with previous validations and demonstrate strong psychometric properties, future studies with larger and more diverse samples are needed to confirm the generalizability of these results. Second, the test–retest interval was relatively short (approximately one week), which may have limited the ability to detect meaningful changes over time and introduced potential recall effects. Finally, while both SPaRQ and HHI are valuable tools for assessing psychosocial aspects of hearing loss, their sensitivity to short-term intervention effects remains uncertain. Future research should explore their responsiveness in longitudinal intervention studies and consider integrating additional measures of psychological flexibility or emotional well-being to capture a more comprehensive picture of patient outcomes.

In the present study, we have limited our analysis to methods based in classical test theory. While there is a strong precedence for this approach, it has been argued that the use of traditional psychometric analysis techniques alone provides weak or circumstantial evidence for the validity of a test [13,29]. More modern methods such as Rasch analysis could be valuable, for example, to refine item-level validity. However, the original English-language questionnaires have already been thoroughly refined and validated with Rasch analysis or related modern methods [9,13]. Our goal was therefore to translate the existing questionnaires, not to further refine them, and as a result, our primary focus was to conduct basic reliability and validity checks. Nevertheless, we recognize that Rasch analysis could still be useful to reveal whether certain items behave differently in the translated version (e.g., due to cultural or linguistic nuances), which might not be obvious through classical test theory methods, as well as that it might highlight the need for minor refinements (e.g., rewording or reordering items) to improve psychometric properties in the new language. Such changes would, however, require additional validation, and therefore, could be a potentially interesting direction for the future.

## 5. Conclusions

In conclusion, this study provides evidence supporting the reliability and construct validity of the Danish translations of the SPaRQ and HHI questionnaires. Both instruments demonstrated strong internal consistency and test–retest reliability, and their subscales were meaningfully correlated, suggesting that they may capture overlapping aspects of the psychosocial impact of hearing loss. While the HHI is a well-established international standard that offers certain advantages, SPaRQ emphasizes social participation—rather than more negatively framed concepts like social isolation—which may be an important distinction in certain contexts, making it a particularly relevant questionnaire in specific applications. Nevertheless, there is a strong overlap between the two PROMs. The lack of correlation between SPaRQ and HHI with audiometric thresholds reinforces the value of patient-reported outcome measures in capturing subjective experiences that are not reflected in objective hearing tests. While the study was limited by a smaller-than-recommended sample size and a short test–retest interval, the findings align with previous validations and underscore the importance of integrating psychosocial assessment into audiological care. Future research should build on these findings by evaluating the responsiveness of these tools in intervention studies and exploring their utility in individualized, holistic rehabilitation planning.

## Figures and Tables

**Table 1:** Demographic characteristics, as well as retention rates.

Characteristic	HA Users	HA Candidates
Enrolled, no.	50	37
Completed		
Round 1 (R1), no. (%)	47 (94)	35 (95)
Total included, no. (%)	47 (94)	32 (86)
Round 2 (R2), no. (%)	44 (88)	29 (78)
Total included, no. (%)	43 (86)	21 (57)
Age in years, mean (SD)	65.7 (11.9)	63.7 (13.8)
Sex, no. (%)		
Female	24 (56)	10 (48)
Male	19 (44)	11 (52)
Other	0 (0)	0 (0)
Prefer not to answer	0 (0)	0 (0)

**Table 2:** Correlation analyses between the SPaRQ and HHI subscales.

	SPaRQ Behaviors	SPaRQ Perceptions
**HHI Emotional**	$r_{s}=0.52$ (p<0.001)	$r_{s}=0.65$ (p<0.001)
**HHI Social**	$r_{s}=0.48$ (p<0.001)	$r_{s}=0.51$ (p<0.001)

**Table 3:** Internal consistency (Cronbach’s alpha) of the subscales in Round 1 (R1), intraclass correlation (ICC) agreement analysis between Round 1 (R1) to Round 2 (R2) (95% confidence intervals indicated in square brackets), and assessments of the magnitude of the individual change from R1 to R2, where the mean and standard deviation (SD) of the change is shown, as well as a 95% confidence interval around the mean. Numbers are reported for the HA users and HA candidate groups combined.

	Cronbach’s Alpha	ICC (R1 vs. R2)	Change (R1 vs. R2)
**SPaRQ Behaviors**	0.91	0.79 [0.67, 0.87]	−2.9 (SD 13.5)
**SPaRQ Perceptions**	0.94	0.83 [0.74, 0.89]	−2.3 (SD 14.7)
**HHI Emotional**	0.86	0.88 [0.80, 0.92]	−0.3 (SD 3.9)
**HHI Social**	0.82	0.80 [0.69, 0.87]	−0.7 (SD 4.4)

## Data Availability

The data presented in this article are not readily available due to data protection. The original contributions presented in this study are, however, included in the article. Further inquiries can be directed to the corresponding author.

## Appendix A

### Appendix A.1

Dette spørgeskema handler om, hvordan din hørenedsættelse påvirker din hverdag. Hvis du normalt bruger høreapparat, skal du svare på spørgsmålene ud fra, hvordan du har det, når du har høreapparat på. For hvert spørgsmål skal du sætte ring om et enkelt tal mellem 0 og 10 for at indikere, hvor besværet du er af din hørenedsættelse:

- Sæt ring om 0, hvis du er helt uenig i det udsagn, der står i skemaet.
- Sæt ring om 10, hvis du er helt enig i det udsagn, der står i skemaet.
- Hvis du laver en fejl, kan du sætte kryds (X) over dit første svar og sætte ring om et nyt tal.

**Table A1:** Danish SPaRQ Section 1.

På grund af min hørenedsættelse synes jeg, at det er svært at:	Helt uenig Helt enig
1. Tale med de ansatte i for eksempel butikker, cafeer og banker.	0 1 2 3 4 5 6 7 8 9 10
2. Deltage i en gruppediskussion eller et gruppemøde.	0 1 2 3 4 5 6 7 8 9 10
3. Dyrke mine fritidsinteresser.	0 1 2 3 4 5 6 7 8 9 10
4. Klare stressende og udfordrende situationer.	0 1 2 3 4 5 6 7 8 9 10
5. Holde ud under lange samtaler.	0 1 2 3 4 5 6 7 8 9 10
6. Mødes med grupper af venner.	0 1 2 3 4 5 6 7 8 9 10
7. Klare mine forpligtelser derhjemme, i mit sociale liv eller på arbejdet.	0 1 2 3 4 5 6 7 8 9 10
8. Følge med i et foredrag eller en forelæsning.	0 1 2 3 4 5 6 7 8 9 10
9. Være med i samtalerne, når jeg mødes med grupper af venner.	0 1 2 3 4 5 6 7 8 9 10

**Table A2:** Danish SPaRQ Section 2.

Min hørenedsættelse betyder, at:	Helt uenig Helt enig
1 Jeg synes sociale arrangementer er stressende.	0 1 2 3 4 5 6 7 8 9 10
2 Jeg føler mig frustreret, når jeg bliver holdt uden for samtaler.	0 1 2 3 4 5 6 7 8 9 10
3 Jeg er bekymret for at gå glip af vigtige lyde eller vigtig information.	0 1 2 3 4 5 6 7 8 9 10
4 Jeg føler mig isoleret under gruppesamtaler.	0 1 2 3 4 5 6 7 8 9 10
5 Jeg orker næsten ikke at deltage i samtaler, hvor jeg har svært ved at høre, hvad der bliver sagt.	0 1 2 3 4 5 6 7 8 9 10
6 Jeg føler mig ensom, selv når jeg er sammen med andre mennesker.	0 1 2 3 4 5 6 7 8 9 10
7 Jeg er bekymret, når jeg skal tale med nogen, jeg ikke kender.	0 1 2 3 4 5 6 7 8 9 10
8 Jeg føler mig isoleret, når jeg mødes med grupper af familie eller venner.	0 1 2 3 4 5 6 7 8 9 10
9 Jeg bliver ked af det, når det er svært for mig at deltage i samtaler.	0 1 2 3 4 5 6 7 8 9 10
10 Jeg synes, at jeg virker dum, når jeg siger noget forkert i en samtale.	0 1 2 3 4 5 6 7 8 9 10

**Table A3:** Danish SPaRQ Section 3.

Cirka hvor ofte bruger du høreapparat. Sæt flueben (✓) i én boks.	
Hver dag ☐ Nogle gange ☐ Aldrig ☐	

## Appendix B

Formålet med denne skala er at identificere, hvilke problemer din hørenedsættelse giver dig. Svar JA, NOGLE GANGE, NEJ, eller IKKE RELEVANT for hvert spørgsmål. Du skal ikke springe et spørgsmål over, selvom det handler om en situation, du undgår på grund af dine problemer med at høre. Hvis du bruger høreapparater, skal du svare ud fra, hvordan du hører uden høreapparater på.

**Table A4:** Danish HHI.

English			Danish				
English HHIE	English HHIA	English RHHI	Danish HHI	Added “not relevant”	RHHI Emotional	RHHI Social	Question
S-1	S-1	11	1			x	Gør problemer med at høre, at du bruger telefonen mindre, end du ellers ville?
E-2	E-2		2				Gør problemer med at høre, at du føler dig pinligt berørt, når du møder nye mennesker?
S-3	S-3	10	3			x	Gør problemer med at høre, at du undgår grupper af mennesker?
E-4	E-4		4				Gør problemer med at høre dig irritabel?
E-5	E-5	4	5		x		Gør problemer med at høre, at du føler dig frustreret, når du taler med familiemedlemmer?
S-6	S-6	2	6			x	Giver høreproblemer dig vanskeligheder, når du er til fest?
E-7			7				Gør problemer med at høre, at du føler dig “dum” eller “uintelligent”?
S-8			8				Har du svært ved at høre, når nogen hvisker?
E-9	E-8	7	9		x		Gør problemer med at høre, at du føler dig handikappet?
S-10	S-9	6	10			x	Giver høreproblemer dig vanskeligheder, når du besøger venner, familie eller naboer?
S-11			11	x			Gør problemer med at høre, at du går mindre til religiøse ceremonier, end du gerne ville?
E-12	E-12	12	12		x		Gør problemer med at høre, at du bliver nervøs?
S-13	S-13	17	13			x	Gør problemer med at høre, at du besøger venner, familie eller naboer mindre, end du gerne ville?
E-14	E-14		14				Gør problemer med at høre, at du har skænderier med familiemedlemmer?
S-15	S-15	1	15			x	Gør problemer med at høre det svært for dig, når du lytter til TV eller radio?
S-16	S-16	18	16			x	Gør problemer med at høre, at du går mindre på indkøb eller shopping, end du gerne ville?
E-17	E-17	3	17		x		Er der overhovedet nogen problemer eller vanskeligheder med din hørelse, der gør dig oprevet eller ked af det?
E-18	E-18	15	18		x		Gør problemer med at høre, at du gerne vil være alene?
S-19	S-19	14	19			x	Gør problemer med at høre, at du taler mindre ofte med familiemedlemmer, end du gerne ville?
E-20	E-20	8	20		x		Føler du, at problemer med din hørelse begrænser eller hæmmer dit personlige eller sociale liv?
S-21	S-21		21				Gør problemer med at høre det svært for dig, hvis du er på restaurant med familie eller venner?
E-22	E-22	16	22		x		Gør problemer med at høre, at du føler dig deprimeret?
S-23	S-23	13	23			x	Gør problemer med at høre, at du lytter mindre ofte til TV eller radio, end du gerne ville?
E-24	E-24	9	24		x		Gør problemer med at høre at du føler dig utilpas, når du taler med venner?
E-25	E-25	5	25		x		Gør problemer med at høre, at du føler dig udenfor, når du er sammen med en gruppe mennesker?
	E-10		26	x			Gør problemer med at høre, at du føler dig frustreret, når du taler med kollegaer, klienter eller kunder?
	S-11		27	x			Giver høreproblemer dig vanskeligheder, når du er i biografen eller teatret?
	S-7		28	x			Giver høreproblemer dig vanskeligheder med at høre/forstå dine kollegaer, klienter eller kunder?
